# A Phase 2 Single-Arm Study of Osimertinib for Radiotherapy-Naive Central Nervous System Metastasis NSCLC: Results for the First-Line Cohort of the OCEAN Study (LOGIK 1603/WJOG 9116L)

**DOI:** 10.1016/j.jtocrr.2023.100587

**Published:** 2023-10-12

**Authors:** Kazushige Wakuda, Hiroyuki Yamaguchi, Hirotsugu Kenmotsu, Minoru Fukuda, Kentaro Ito, Yuko Tsuchiya-Kawano, Kentaro Tanaka, Taishi Harada, Yuki Nakatani, Satoru Miura, Toshihide Yokoyama, Tomomi Nakamura, Miiru Izumi, Atsushi Nakamura, Satoshi Ikeda, Koichi Takayama, Kenichi Yoshimura, Kazuhiko Nakagawa, Nobuyuki Yamamoto, Kenji Sugio

**Affiliations:** a Division of Thoracic Oncology, Shizuoka Cancer Center Hospital, Shizuoka, Japan; b Department of Respiratory Medicine, Nagasaki University Graduate School of Biomedical Sciences, Nagasaki, Japan; c Cancer Treatment Center, Nagasaki Prefecture Shimabara Hospital, Shimabara, Japan; d Department of Respiratory Medicine, Matsusaka Municipal Hospital Respiratory Center, Mie, Japan; e Department of Respiratory Medicine, Kitakyushu Municipal Medical Center, Fukuoka, Japan; f Research Institute for Diseases of the Chest, Graduate School of Medical Sciences, Kyushu University, Fukuoka, Japan; g Department of Respiratory Medicine, Japan Community Health Care Organization (JCHO) Kyushu Hospital, Fukuoka, Japan; h Department of Medical Oncology, Osaka City General Hospital, Osaka, Japan; i Department of Internal Medicine, Niigata Cancer Center Hospital, Niigata, Japan; j Department of Respiratory Medicine, Kurashiki Central Hospital, Okayama, Japan; k Division of Hematology, Respiratory Medicine and Oncology, Department of Internal Medicine, Faculty of Medicine, Saga University, Saga, Japan; l Department of Respiratory Medicine, National Hospital Organization, Omuta National Hospital, Fukuoka, Japan; m Department of Pulmonary Medicine, Sendai Kousei Hospital, Miyagi, Japan; n Department of Respiratory Medicine, Kanagawa Cardiovascular and Respiratory Center, Kanagawa, Japan; o Department of Pulmonary Medicine, Graduate School of Medical Science, Kyoto Prefectural University of Medicine, Kyoto, Japan; p Center for Integrated Medical Research, Hiroshima University Hospital, Hiroshima University, Hiroshima, Japan; q Department of Medical Oncology, Kindai University Faculty of Medicine, Osaka, Japan; r Internal Medicine III, Wakayama Medical University, Wakayama, Japan; s Department of Thoracic and Breast Surgery, Oita University Faculty of Medicine, Oita, Japan

**Keywords:** Non–small cell lung cancer, First-line, CNS metastasis, Brain metastasis, Osimertinib

## Abstract

**Introduction:**

Osimertinib may be effective in treating central nervous system (CNS) metastasis, but its efficacy in treating radiation therapy (RT)-naive metastasis is unclear. The OCEAN study assessed the efficacy of osimertinib against RT-naive CNS metastasis in patients previously treated (T790M cohort) and untreated patients (first-line cohort) with *EGFR* mutation. Here, we report the results of the first-line cohort.

**Methods:**

Previously untreated patients with RT-naive CNS metastasis and *EGFR* mutation-positive NSCLC were treated with osimertinib. The brain metastasis response rate (BMRR), progression-free survival (PFS), and overall survival in the first-line cohort were secondary end points.

**Results:**

A total of 26 patients were enrolled in the study between September 2019 and July 2020. The median age was 72.0 years with 80.8% female. There were 20 patients who had multiple CNS metastases. BMRR assessed by PAREXEL criteria was 76.9% (90% confidence interval [CI]: 63.3%–90.5%), BMRR assessed by Response Evaluation Criteria in Solid Tumors was 76.9% (95% CI: 54.0%–99.8%), and median PFS of CNS metastasis was 22.0 months (95% CI: 9.7 mo–not reached). The overall response rate was 64.0% (95% CI: 45.2%–82.8%), median PFS was 11.5 months (95% CI: 6.9 mo–not reached), and median survival time was 23.7 months (95% CI: 16.5 mo–not reached). Paronychia and increased creatinine level were the most frequent nonhematological toxicities observed in 13 patients (50%). Grade three and higher adverse events were less than 10%, and there were no treatment-related deaths. Pneumonitis was observed in five patients (19.2%).

**Conclusions:**

These results suggest that osimertinib is effective in untreated patients with RT-naive asymptomatic CNS metastasis in a clinical practice first-line setting.

**Trial registration:**

UMIN identifier: UMIN000024218. jRCT identifier: jRCTs071180017.

## Introduction

It has been reported that 15% to 30% of patients with NSCLC harboring activating *EGFR* mutations have brain metastasis (BM) at diagnosis.^1^, ^2^, ^3^ Because the central nervous system (CNS) has a blood-brain barrier, BM are less effective than other metastatic sites for chemotherapy. Although radiation therapy (RT), such as whole-brain radiation therapy (WBRT) and stereotactic radiation therapy, is a standard treatment for CNS metastasis, the median survival time of patients receiving WBRT is only 4 to 8 months.^4^^,^^5^ It has also been reported that WBRT increases the risk of cognitive dysfunction.

Osimertinib is one of the standard treatments for patients with NSCLC harboring *EGFR* mutations, based on the AURA3 and FLAURA study.^6^^,^^7^ In a preclinical model, osimertinib was found to have greater penetration into the CNS than other *EGFR* -tyrosine kinase inhibitors (TKIs).^8^ Some subgroup analyses of clinical trials found the efficacy of osimertinib in patients harboring *EGFR* mutations with BM.^9^, ^10^, ^11^ Nevertheless, these reports included patients with BM treated with RT. We designed the OCEAN study to assess the efficacy of osimertinib in patients with RT-naive CNS metastasis NSCLC because the efficacy of osimertinib for BM not treated with RT was unclear.^12^ The OCEAN study included the following two cohorts: the T790M cohort and the first-line cohort. The primary end point of the OCEAN study was the BM response rate (BMRR) assessed by the PAREXEL criteria in the T790M cohort, and we previously reported the results of the T790M cohort.^13^ The BMRR of the T790M cohort was 66.7% (90% confidence interval [CI]: 54.3%–79.1%), and the T790M cohort met the primary end point. The median cerebrospinal fluid (CSF) penetration rates of osimertinib and AZ5104 were 0.79% and 0.53%, respectively. The results of the T790M cohort study revealed the efficacy of osimertinib in patients harboring *EGFR* T790M mutations with RT-naive CNS metastasis. Here, we report the results of a first-line cohort study.

## Material and Methods

### Study Design and Patients

As previously described, the OCEAN study was a multicenter, single-arm phase II study.^12^ In the first-line cohort, the inclusion criteria were as follows: patients with a histologically or cytologically confirmed diagnosis of NSCLC; confirmed *EGFR* mutations (*EGFR* exon 19 deletion or exon 21 L858R point mutation); previously untreated with *EGFR* TKIs; stage IV or postoperative relapse; patients with BM of 5 mm or more in the long axis; patients who had undergone no previous RT for BM; and an Eastern Cooperative Oncology Group performance status of 0 to 2. Patients with symptomatic BM requiring RT, surgical resection, or active double cancer were excluded. Patients with interstitial lung disease, drug-induced interstitial lung disease, or radiation pneumonitis requiring treatment with steroids were also excluded. Before registration in this trial, contrast-enhanced computed tomography (CT) scanning of the chest and abdomen and contrast-enhanced magnetic resonance imaging (MRI) of the brain with a slice thickness of less than 3 mm were required. CT and MRI scans were performed every 6 weeks within 1 year of the date of registration and every 3 months thereafter. Osimertinib (80 mg, once daily) was administered until progression, death, unacceptable adverse events, or withdrawal of consent to participate in the study.

All patients provided written informed consent before the study enrollment.

### Statistical Analysis

The primary end point of the OCEAN study was BMRR, assessed using the PAREXEL criteria in the T790M cohort. BMRR, overall response rate (ORR), progression-free survival (PFS), and overall survival (OS) in the first-line cohort were secondary end points. The PAREXEL criteria are tools for assessing BM and have recently been used in many trials. According to these criteria, a target lesion of BM was defined as having a size of 5 mm or more along the long axis, and a maximum of five lesions were selected to calculate the sum of the diameters. Nontarget lesions were defined as lesions less than 5 mm in size. There was no limit to the number of nontarget lesions. The assessment of BM response was similar to that used in the RANO response assessment criteria for BM and Response Evaluation Criteria in Solid Tumors (RECIST) criteria.^14^^,^^15^ A complete response was the absence of all lesions, and the entire brain is assessable. A partial response (PR) was defined as at least a 30% decrease in the sum of the diameters. Progressive disease (PD) was defined as at least a 20% increase in the sum of the diameters. Stable disease was defined as neither growth sufficient to qualify for PD nor response sufficient to qualify for PR. Considering the threshold value and expected value of BMRR of 55% and 80% in the first-line cohort, the sample size of the first-line cohort was calculated to be 25 considering dropout patients at one-sided alpha = 0.05 and power = 0.8.

The OCEAN study was conducted in compliance with the principles of the Declaration of Helsinki and approved by the Central Review Board of the Clinical Research Network at Fukuoka. This trial was registered in the University Hospital Medical Information Network Trials Registry (UMIN000024218) and the Japan Registry of Clinical Trials (jRCTs071180017).

### Translational Research

To investigate the relationship between the concentration of osimertinib and the treatment effect, we assessed the blood concentration of osimertinib on day 22, which was considered to represent a steady state.^16^ The blood concentration of osimertinib was measured using the plasma samples. The blood samples were collected 22 days after osimertinib administration. In addition to analyzing the blood concentration of osimertinib, we analyzed the concentration of AZ5104, an active osimertinib metabolite.^17^ We also determined the CSF concentrations of osimertinib and AZ5104 to analyze their penetration into the fluid. The blood concentration of osimertinib was also assessed at the time of CSF collection. The CSF was collected if possible. Blood and CSF concentrations of osimertinib were assessed using HB-13-050 and HB-13-081 (HPLC-MS/MS). To determine whether the *EGFR* C797S mutation was present before progression, we also evaluated tumor-derived DNA for *EGFR* mutations by digital polymerase chain reaction, including the C797S point mutation, in plasma specimens. Plasma specimens for *EGFR* mutation analysis were collected three times, as follows: before treatment, 22 days after osimertinib administration, and on the date of diagnosis of the progressive disease. In the first-line cohort, we also evaluated tumor-derived DNA by Guardant360 liquid biopsy (Guardant360 CDX LDT 2.11, Guardant Health, Redwood City, CA) for gene alterations in plasma specimens to simultaneously analyze the mechanism of acquired resistance to osimertinib and the *EGFR* mutation status. Guardant360 can detect point mutations and deletion or insertion variants in 74 genes, amplification in 18 genes, and fusion in six genes. For single-nucleotide variants, insertions and deletions, and fusions, the cutoff for mutant variants was more than or equal to 0.04%, and for amplifications, it was more than or equal to 2.18 copies.

## Results

### Patient Characteristics

A total of 26 patients from 14 institutions were enrolled between September 2019 and July 2020. The data cutoff date was January 21, 2022. There were no ineligible patients, and all patients were included in the evaluation of the treatment efficacy. There were 13 patients included in the evaluation of the BMRR using the RECIST criteria, and 25 patients were included in the evaluation of the ORR (Supplementary Fig. 1). Patient characteristics are found in Table 1. The median age was 72 years; 80.8% of the patients were female; 73.1% of the patients had stage IV at diagnosis; only two patients had an Eastern Cooperative Oncology Group performance status of 2; and nine patients were current or former smokers. Almost all patients (96.2%) were histologically diagnosed with adenocarcinoma. Furthermore, 15 patients (57.7%) had *EGFR* exon 19 deletions, whereas the rest had L858R point mutations. Regarding the status of CNS metastasis, 20 patients had multiple metastases and four patients had symptomatic CNS metastasis. The median baseline CNS target lesion size was 8.7 mm (range 5.0–49.6 mm).

**Table 1:** Patient Characteristics of the First-Line Cohort

Characteristics		N = 26
Age, y	Median	72.0
	Range	55–82
Gender, n (%)	Male	5 (19.2)
	Female	21 (80.8)
Stage at diagnosis, n (%)	IV	19 (73.1)
	Postoperative	7 (26.9)
ECOG performance status, n (%)	0	9 (34.6)
	1	15 (57.7)
	2	2 (7.7)
Histological type, n (%)	Adenocarcinoma	25 (96.2)
	Others	1 (3.8)
Smoking history, n (%)	Current or former	9 (34.6)
	Never	17 (65.5)
*EGFR* mutation type, n (%)	Exon 19 deletion	15 (57.7)
	L858R	11 (42.3)
CNS lesion, n (%)	Single	6 (23.1)
	Multiple	20 (76.9)
Symptomatic CNS metastasis, n (%)	Present	4 (15.4)
	Absent	21 (80.8)
	Unknown	1 (3.8)
Baseline CNS target lesion size	Median	8.7 mm
	Range	5.0–49.6 mm

CNS, central nervous system; ECOG, Eastern Cooperative Oncology Group.

### Efficacy

**Figure 1:**
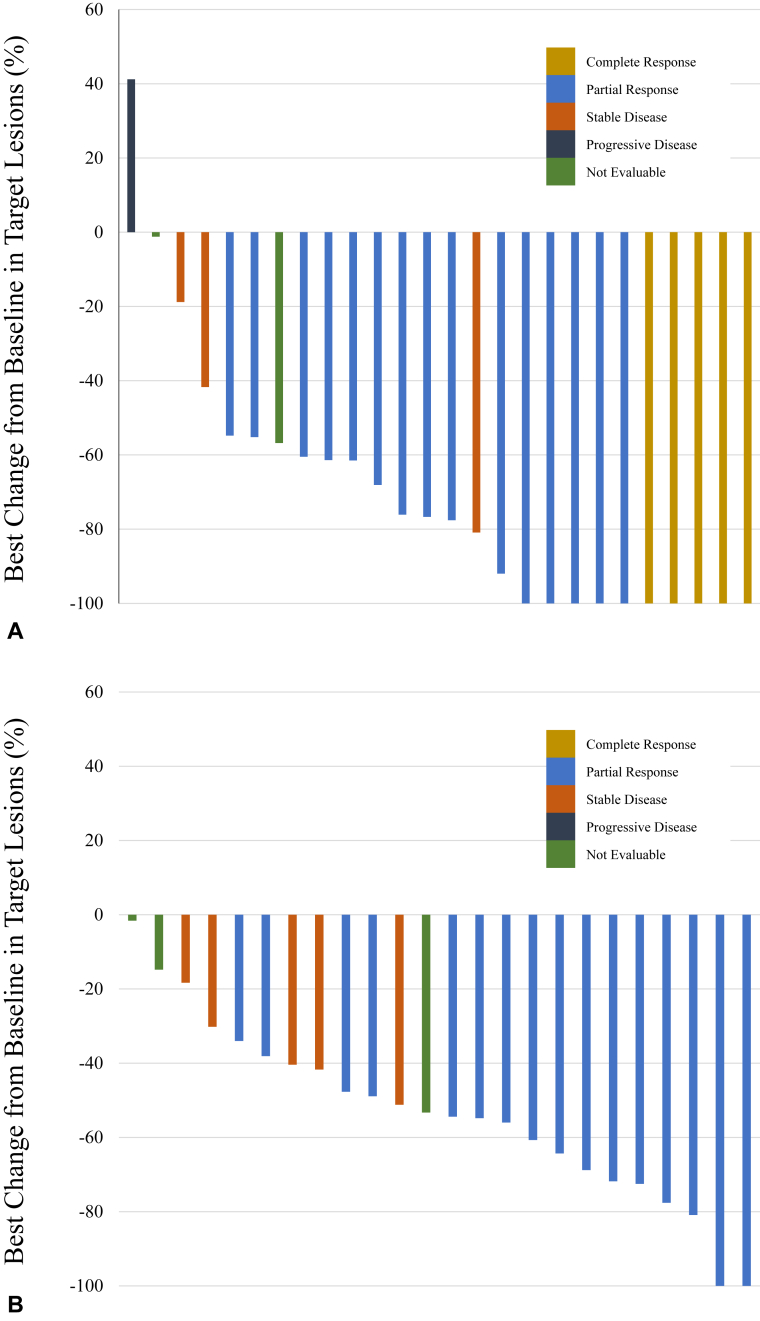
Best target-lesion responses. (*A*) BM response assessed by the PAREXEL criteria (N = 26). (*B*) Overall response (N = 25). BM, brain metastasis.

**Figure 2:**
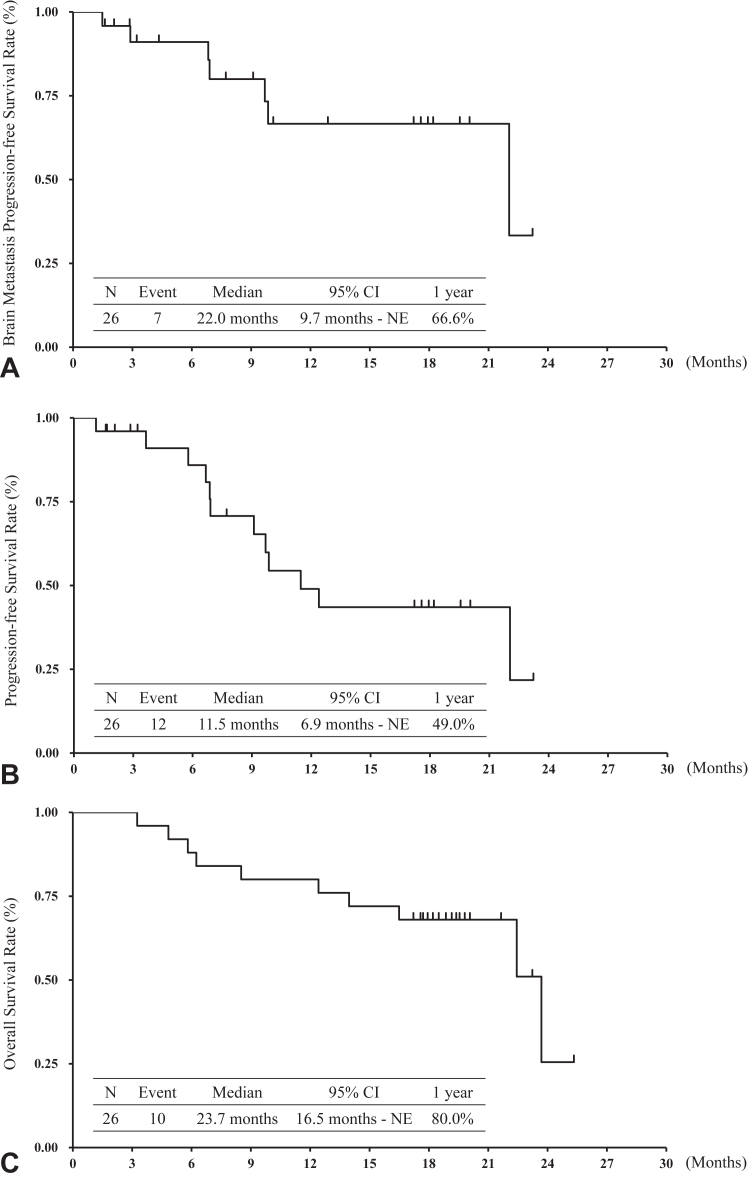
BM results (N = 26). (*A*) overall survival; (*B*) progression-free survival; (*C*) progression-free survival of BM. BM, brain metastasis.

**Figure 3:**
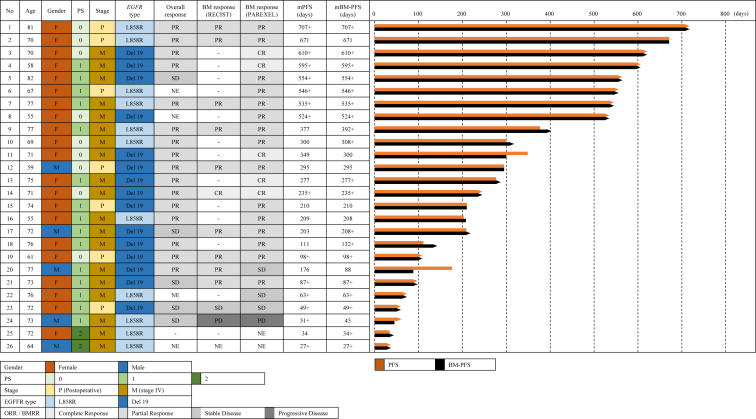
Swimmer plot. BM, brain metastasis; CR, complete response; F, female; M, male; mBM-PFS, median brain metastasis progression-free survival; mPFS, median progression-free survival; NE, not evaluable; PD, progressive disease; PR, partial response; PS, performance status; SD, stable disease.

The BMRR assessed using the PAREXEL criteria was 76.9% (90% CI: 63.3%–90.5%), and five patients (19.2%) had complete response in all 26 patients (Table 2 and Fig. 1*A*). Because the lower limit of the 90% CI in the BMRR assessed using the PAREXEL criteria was more than 55%, statistical significance was observed. The BMRR assessed by the RECIST criteria was 76.9% (95% CI: 54.0%–99.8%) in 13 patients, and the ORR was 64.0% (95% CI: 45.2%–82.8%) in 25 patients (Table 2 and Fig. 1*B*). At the data cutoff, protocol treatment had been completed in 19 patients and was still ongoing in seven patients. The main reasons for treatment discontinuation were disease progression in 10 patients (38.5%) and adverse events in five patients (19.2%). The number of BM-PFS events was seven, and the median BM-PFS was 22.0 months (95% CI: 9.7 mo–not evaluable) (Fig. 2*A*). The one-year BM-PFS rate was 66.6%. Five of 19 patients who discontinued osimertinib treatment received RT for BM. The number of PFS events was 12. The median PFS and one-year PFS rate were 11.5 months (95% CI: 6.9 mo‒not evaluable) and 49.0%, respectively (Fig. 2*B*). The number of OS events was 10. The median survival time and one-year survival rate were 23.7 months (95% CI: 16.5–not evaluable) and 80.0%, respectively (Fig. 2*C*). Figure 3 illustrates the swimmer plots of PFS and BM-PFS.

**Table 2:** Response Rate

Variable	BMRR Is Assessed by PAREXEL Criteria	BMRR Is Assessed by RECIST Criteria	ORR Is Assessed by RECIST Criteria
n	26	13	25
Response, n (%)			
CR	5 (19.2)	1 (7.7)	0 (0)
PR	15 (57.7)	9 (69.2)	16 (64.0)
SD	3 (11.5)	1 (7.7)	5 (20.0)
PD	1 (3.8)	1 (7.7)	0 (0)
NE	2 (7.7)	1 (7.7)	4 (16.0)
Response rate	76.9%	76.9%	64.0%
90% CI	63.3%–90.5%		
95% CI		54.0%–99.8%	45.2%–82.8%

BMRR, brain metastasis response rate; CI, confidence interval; CR, complete response; NE, not evaluable; ORR, overall response rate; PD, progressive disease; PR, partial response; RECIST, Response Evaluation Criteria in Solid Tumors; SD, stable disease.

### Toxicities

Adverse events are found in Table 3. Although hematological toxicities of any grade were observed in approximately 20% to 60% of patients, there was only one case of grade 3 neutropenia. Paronychia and increased creatinine level were the most frequent nonhematological toxicities observed in 13 patients (50%), followed by stomatitis (46.2%), diarrhea (46.2%), increased aspartate transaminase level (42.3%), anorexia (38.5%), acneiform rash (34.6%), increased alanine transaminase level (30.8%), and dry skin (30.8%). All grade three or higher adverse events were observed in less than 10% of the patients, and there were no treatment-related deaths. Pneumonitis was observed in five patients, which led to treatment discontinuation. Nevertheless, all pneumonitis cases were of grade two or lower. Dose reduction and interruption was required in six and 10 patients, respectively. The reasons for dose reduction were as follows: diarrhea in two, pruritus in one, anorexia in one, stomatitis in one, and skin disorder in one patient.

**Table 3:** Adverse Events

Variable	Any Grade, n (%)	Grade ≥3, n (%)
Hematologic		
Leukopenia	11 (42.3)	0 (0)
Neutropenia	6 (23.1)	1 (3.8)
Anemia	15 (57.7)	0 (0)
Thrombocytopenia	12 (46.2)	0 (0)
Nonhematologic		
Paronychia	13 (50.0)	0 (0)
Creatinine increased	13 (50.0)	0 (0)
Stomatitis	12 (46.2)	2 (7.7)
Diarrhea	12 (46.2)	1 (3.8)
AST increased	11 (42.3)	0 (0)
Anorexia	10 (38.5)	3 (11.5)
Rash acneiform	9 (34.6)	0 (0)
ALT increased	8 (30.8)	1 (3.8)
Dry skin	8 (30.8)	0 (0)
Fatigue	7 (26.9)	0 (0)
Pruritis	6 (23.1)	1 (3.8)
Nausea	6 (23.1)	0 (0)
Pneumonitis	5 (19.2)	0 (0)

ALT, alanine aminotransferase; AST, aspartate aminotransferase.

### Biomarker analysis

**Figure 4:**
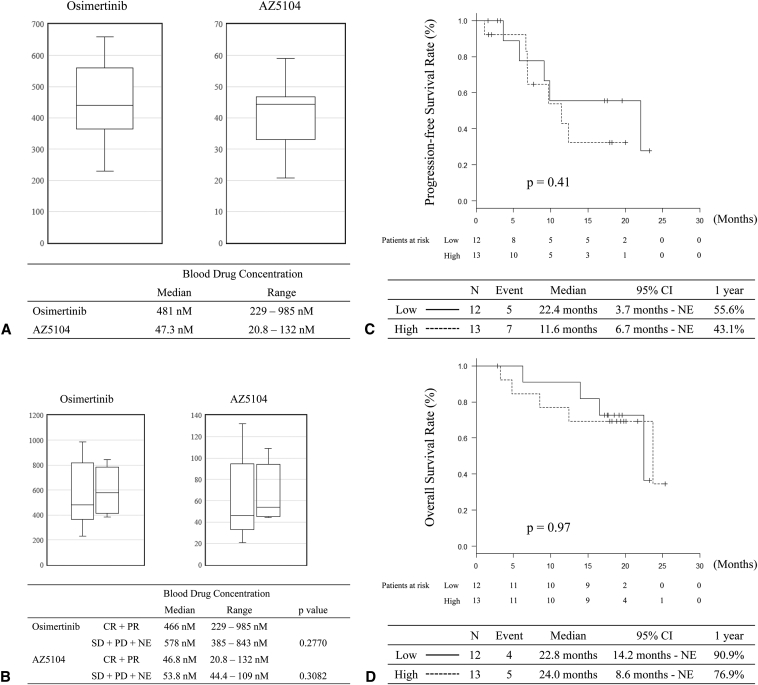
Drug concentration. (*A*) Blood trough concentration of osimertinib and AZ5104; (*B*) blood trough concentration of osimertinib and AZ5104 according to BMRR assessed by PAREXEL criteria; (*C*) progression-free survival separated by blood osimertinib concentration (high ≥ 481 nM); (*D*) overall survival separated by blood osimertinib concentration (high ≥ 481 nM). BMRR, brain metastasis response rate.

Blood drug concentrations were analyzed in 26 patients who were assessed for the efficacy of osimertinib against CNS metastasis. The trough concentrations of osimertinib and AZ5104 on day 22 are found in Figure 4*A*. The median blood concentrations of osimertinib and AZ5104 were 481 nM (range, 229–985 nM) and 47.3 nM (range: 20.8–132 nM), respectively. The BMRR assessed by the PAREXEL criteria and day 22 trough concentrations of osimertinib and AZ5104 are found in Figure 4*B*. The median blood concentrations of osimertinib and AZ5104 were 466 nM (range: 229–985 nM) and 46.8 nM (range: 20.8–132 nM) in patients with CNS responses (CR and PR). Nevertheless, median blood concentrations of osimertinib and AZ5104 were 578 nM (range: 385–843 nM) and 53.8 nM (range: 44.4–109 nM) in patients with CNS nonresponse (SD + PD + NE). There was no significant correlation between BMRR and drug concentration (*p* = 0.2770 for osimertinib and *p* = 0.3082 for AZ5104). In addition, we divided the participants into low- and high-concentration groups based on the median blood drug concentration. There was no relationship between PFS or OS and the drug concentration (Fig. 4*C* and *D*). Although we planned to analyze the CSF, no CSF was collected from the patients during the study period. Analysis of the CSF drug concentration was not performed in the first-line cohort.

Blood samples to evaluate *EGFR* mutations were obtained from 24 patients before treatment, 25 on day 22, and 10 at disease progression. The T790M and C797S point mutations were not detected before the treatment. On day 22, the T790M point mutation was detected in one of the 25 samples (4%). C797S was detected in one of the 10 samples (10%) during disease progression.

**Figure 5:**
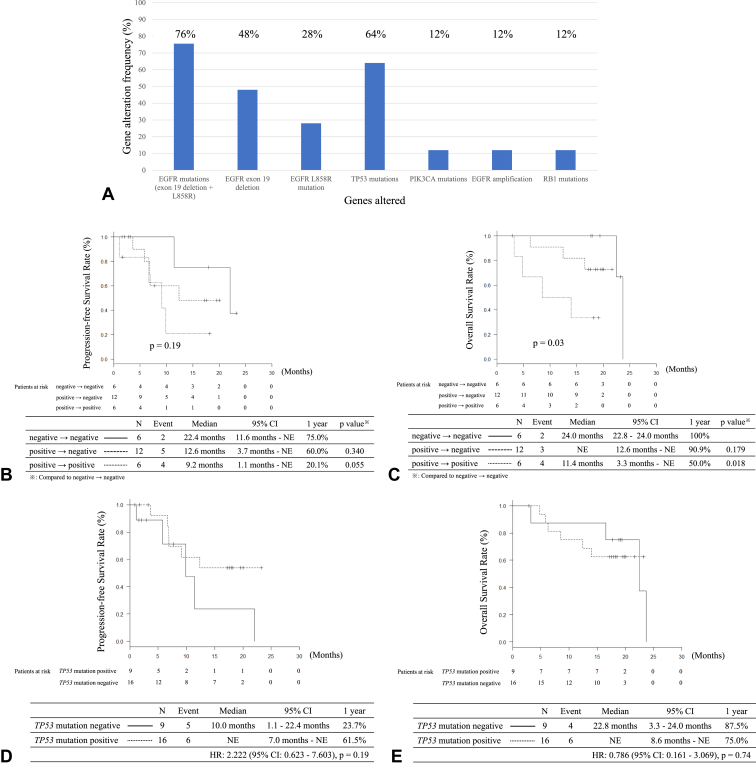
Analysis of gene alterations using Guardant360. (*A*) Frequency of gene alterations before treatment; (*B*) progression-free survival according to the change of positivity of *EGFR* mutations between before treatment and day 22; (*C*) overall survival according to the change of positivity of *EGFR* mutations between before treatment and day 22; (*D*) progression-free survival according to the *TP53* mutations; (*E*) overall survival according to the *TP53* mutations.

There were 25 samples analyzed using Guardant360 before the treatment, and 24 samples were analyzed on day 22. Only nine samples were analyzed for disease progression because many patients did not experience disease progression at the end of the study. The results of the gene alterations analyzed by Guardant360 before the treatment are found in Figure 5*A*. *TP53* mutations were detected in 16 (64%) samples. *EGFR* exon 19 deletions and L858R mutations were found in 12 (48%) and seven (28%) samples, respectively. Guardant360 detected *EGFR* exon 19 deletions in 12 of the 15 (80%) patients with *EGFR* exon 19 deletions. In contrast, *EGFR* L858R mutations were detected in seven of 11 patients (64%). On day 22, 18 of the 19 samples with *EGFR* mutations were analyzed, and 12 of the 18 samples (67%) were negative for *EGFR* mutations. The results of PFS and OS analyses according to the pattern of *EGFR* mutation change are found in Figure 5*B* and *C*. There was no significant difference in PFS between the three groups: *EGFR* mutation negative before the treatment, *EGFR* mutation positive to negative, and *EGFR* mutation positive to positive (*p* = 0.19). Nevertheless, a significant difference was observed in OS between the three groups (*p* = 0.03). The negative-to-negative group had a significantly longer OS than the positive-to-positive group (*p* = 0.018). In the case of patients with *TP53* mutations before the treatment, there was no significant difference in PFS and OS between patients with *TP53* positivity and negativity (Fig. 5*D* and *E*).

## Discussion

Although previous reports have revealed the efficacy of osimertinib in patients with NSCLC with CNS metastases, these reports involved exploratory subgroup analyses, and the efficacy of osimertinib in patients with previously untreated CNS metastases remains unclear. To our knowledge, the OCEAN study is the first prospective trial to evaluate the efficacy of osimertinib in patients with NSCLC with untreated BM.

A subgroup analysis of osimertinib efficacy in patients with CNS metastasis was reported in the FLAURA study, which compared osimertinib with gefitinib or erlotinib in previously untreated patients with *EGFR* mutations.^11^ This study included 128 patients with measurable or nonmeasurable CNS metastasis (cFAS: osimertinib, n = 61; gefitinib or erlotinib, n = 67) and 41 patients with measurable CNS metastasis (cEFR: osimertinib, n = 22; gefitinib or erlotinib, n = 19). In patients treated with osimertinib, the overall CNS responses to cFAS and cEFR were 66% and 91%, respectively. The BMRR assessed using the PAREXEL criteria was 76.9%, and the BMRR assessed using the RECIST criteria was 76.9% in the OCEAN study. The BMRR of the OCEAN study tended to be inferior to that of the subgroup analysis of the FLAURA study. Nevertheless, the CNS progression-free rate at 12 months was approximately 80% in the subgroup analysis of the FLAURA study and in the OCEAN study. RT for CNS metastasis might affect the efficacy of osimertinib for CNS response because subgroup analysis of the FLAURA study included patients treated with RT for CNS metastasis, and these patients accounted for 25%.

A recent retrospective study revealed that there was no significant difference in time to progression and time to intracranial progression between patients treated with new-generation TKI alone and those treated with TKI plus RT in patients with CNS metastases harboring *EGFR* mutations or *ALK* rearrangement.^18^ This report also revealed that the time to intracranial progression was longer than the time to progression in TKI alone and TKI plus RT. In the OCEAN study, the PFS of patients with BM was also longer than that of the general population. These results suggest that control of extracranial tumors might be a more important factor for survival in the era of new-generation TKIs.

The toxicities of osimertinib observed in the OCEAN study were comparable with those reported previously, except for pneumonitis. Although all pneumonitis was grade two or lower, pneumonitis was observed in five patients (19.2%). In the FLAURA study, interstitial lung disease was reported in 4% of the patients treated with osimertinib.^7^ In the Japanese subset of the FLAURA study, pneumonitis was observed in eight of the 65 patients (12.3%).^19^ The WJOG9717L study, which compared osimertinib monotherapy with osimertinib plus bevacizumab in previously untreated Japanese patients harboring *EGFR* mutations, revealed that 18.3% of the patients experienced pneumonitis.^20^ Furthermore, real-world data from Japanese patients treated with osimertinib revealed that 12.8% of patients developed all-grade pneumonitis.^21^ These reports suggest that pneumonitis may be more common in Japanese patients than in other populations; however, the reasons for this remain unclear.

In the first-line cohort, there was no significant relationship between the BMRR assessed using the PAREXEL criteria and drug concentration. We also reported that blood drug concentration did not affect the efficacy of osimertinib in the T790M cohort.^13^ In patients with NSCLC harboring *EGFR* mutations, Kenmotsu et al.^22^ reported no relationship between response and total and unbound erlotinib exposure. The BLOOM study that assessed the efficacy of osimertinib 160 mg in patients with CNS metastasis reported an intracranial ORR of 55.0%, although all patients had leptomeningeal metastasis in this study.^23^ These results, including the OCEAN study, suggest that the blood concentration of TKI might not affect efficacy in patients with NSCLC harboring *EGFR* mutations. With respect to toxicities, grade three or higher adverse events were observed in 66% of the patients in the BLOOM study and in less than 10% of the patients in the OCEAN study. These results suggest that an 80-mg dose of osimertinib may be feasible for patients with asymptomatic RT-naive CNS metastasis.

Guardant360 is a qualitative next-generation sequencing method that uses blood samples. Recently, some studies have assessed the utility of Guardant360 for gene analysis in patients with NSCLC. Cho et al.^24^ reported *EGFR* driver mutations in 48.6% of East Asian blood samples analyzed by Guardant360. In cases other than Asian, *EGFR* driver mutations were detected in approximately 20% to 25% of samples by Guardant360.^25^, ^26^, ^27^ Furthermore, the NILE study revealed that Guardant360 was noninferior to physician discretion tissue genotyping in the detection rate of gene alterations (Guardant360, 27.3%, versus tissue genotyping, 21.3%).^27^ Because it was reported that approximately 30% of tumor samples were insufficient or inadequate for genomic profiling by next-generation sequencing, these reports suggested that Guardant360, which uses blood samples, might be an effective method for the analysis of gene alterations in patients with insufficient tissue samples or patients with tissue that is difficult to use in a biopsy.^28^^,^^29^ Tran et al.^30^ reported no significant differences in the efficacy of *EGFR* TKIs between patients treated based on tumor-based comprehensive profiling and those treated based on Guardant360. Furthermore, the NILE study revealed that the ORR and disease control rates of the patients treated with targeted therapy based on the results of Guardant360 were 58% and 94%, respectively.^31^ In the OCEAN study, the results of gene alterations analyzed by Guardant360 revealed that 19 of the 25 samples (76%) had *EGFR* mutations. Because some patients with CNS metastasis are in poor condition and difficult to have a biopsy, the Guardant360 might offer treatment opportunities for these patients.

The first-line cohort of the OCEAN study had several limitations. First, the OCEAN study was a single-arm phase II study, as previously mentioned. The OCEAN study did not compare osimertinib alone with osimertinib plus RT. Nevertheless, the OCEAN study evaluated the efficacy of osimertinib in patients with RT-naive CNS metastasis because we excluded patients with previously treated CNS metastasis to eliminate the effects of RT. Few prospective studies have assessed the efficacy of osimertinib in patients with RT-naive CNS metastases. We believe that the OCEAN study would be useful for previously untreated patients with CNS metastasis because it suggested that WBRT might be avoided by osimertinib in patients with CNS metastasis. Second, the first-line cohort had a small number of patients and was the secondary end point. Although the secondary end point was not based on sample size calculation in general, the sample size of the OCEAN study was calculated considering the threshold value and expected value of the BMRR of 55% and 80%, respectively. The BMRR of the first-line cohort was statistically significant, and we thought that the results were clinically meaningful. Finally, we did not analyze the efficacy of osimertinib for leptomeningeal metastasis. Leptomeningeal metastasis is an important issue in patients harboring *EGFR* mutations.

In conclusion, the OCEAN study results suggest that osimertinib is effective for untreated patients with asymptomatic RT-naive CNS metastasis in a first-line clinical practice setting. Considering late toxicities caused by radiotherapy against CNS metastases, osimertinib administration before RT might be a better treatment strategy for patients with NSCLC harboring *EGFR* mutations and RT-naive CNS metastases. The results of the pharmacokinetic analysis revealed no correlation between blood drug concentration and treatment efficacy.

## CRediT Authorship Contribution Statement

**Kazushige Wakuda:** Conceptualization, Investigation, Resources, Writing—original draft.

**Hiroyuki Yamaguchi:** Conceptualization, Investigation, Resources, Writing—review and editing.

**Hirotsugu Kenmotsu:** Conceptualization, Funding acquisition, Investigation, Writing—review and editing.

**Minoru Fukuda:** Conceptualization, Funding acquisition, Investigation, Writing—review and editing.

**Kentaro Ito:** Resources, Writing—review and editing.

**Yuko Tsuchiya-Kawano:** Resources, Writing—review and editing.

**Kentaro Tanaka:** Resources, Writing—review and editing.

**Taishi Harada:** Resources, Writing—review and editing.

**Yuki Nakatani:** Resources, Writing—review and editing.

**Satoru Miura:** Resources, Writing—review and editing.

**Toshihide Yokoyama:** Resources, Writing—review and editing.

**Tomomi Nakamura:** Resources, Writing—review and editing.

**Miiru Izumi:** Resources, Writing—review and editing.

**Atsushi Nakamura:** Resources, Writing—review and editing.

**Satoshi Ikeda:** Resources, Writing—review and editing.

**Koichi Takayama:** Resources, Writing—review and editing.

**Kenichi Yoshimura:** Formal Analysis, Writing—review and editing.

**Kazuhiko Nakagawa:** Supervision, Writing—review and editing.

**Nobuyuki Yamamoto:** Supervision, Writing—review and editing.

**Kenji Sugio:** Supervision, Writing—review and editing.
