# Supplementary figures and images for: A Phase 2 Single-Arm Study of Osimertinib for Radiotherapy-Naive Central Nervous System Metastasis NSCLC: Results for the First-Line Cohort of the OCEAN Study (LOGIK 1603/WJOG 9116L)
Source: JTO Clin Res Rep. 2023 Oct 12;4(12):100587. doi: 10.1016/j.jtocrr.2023.100587 (PMC10689268; doi:10.1016/j.jtocrr.2023.100587)

## Supplementary Figure 1

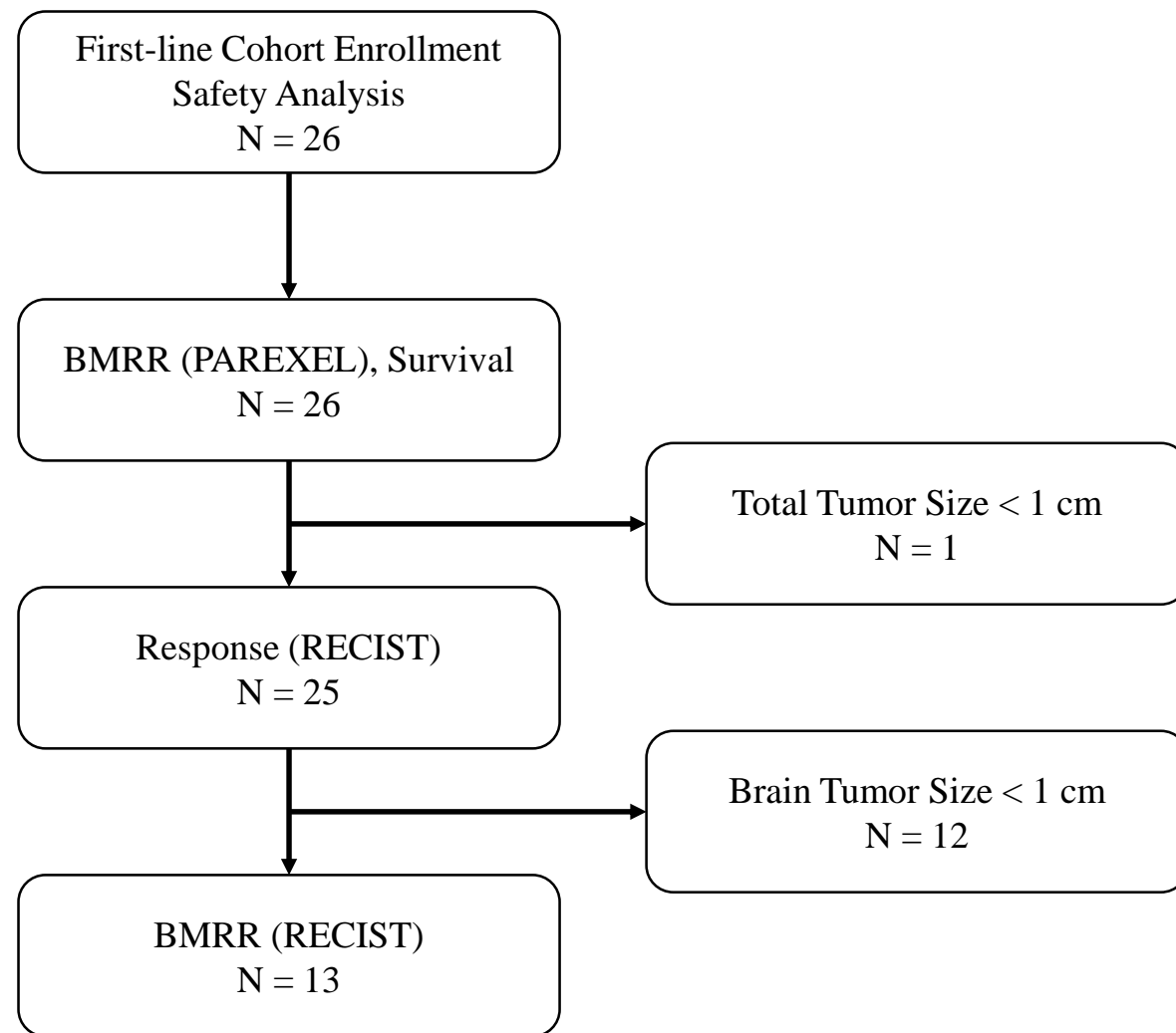

Supplement: Supplementary Figure [file mmc1.pdf]
